# Is Volunteering an Equalizer? A Quasi-Experimental Study Using Propensity Score Analysis

**DOI:** 10.1093/geroni/igaa057.117

**Published:** 2020-12-16

**Authors:** Seoyoun Kim, Cal Halvorsen

**Affiliations:** 1 Texas State University, San Marcos, Texas, United States; 2 Boston College, Chestnut Hill, Massachusetts, United States

## Abstract

Formal volunteering in later life has been extolled as beneficial for both physical and mental health. However, research points to potential selection bias, in that older adults with key advantages, such as higher wealth, are more likely to volunteer and reap its benefits. As such, we test two competing propositions: Volunteering may act as an equalizer if it benefits the health of the least wealthy the most, or it may further exacerbate disparities if it benefits the health of the wealthiest the most. To that end, we analyzed data from the 2012 and 2014 waves of the Health and Retirement Study (N≈15,000). First, we used relevant covariates (e.g., sociodemographic characteristics, informal volunteering, and health) in 2012 to predict volunteering in 2014, developing the propensity score weights from these results. We then performed several regression analyses to assess the influence of volunteering on self-reported health and depressive symptoms among the general population (ATE) and volunteers themselves (ATT), while comparing the findings for the highest and lowest wealth quintiles. We found that volunteering enhanced self-reported health and reduced depressive symptoms. Further, those in the highest quintile experienced significantly fewer depressive symptoms from volunteering while those in the lowest quintile did not, albeit with no significant differences between the two coefficients. The study enhances the nuanced understanding of volunteering and health while suggesting that unmeasured factors felt strongest among the least wealthy—such as financial distress, discrimination, or lack of organizational support—may attenuate the benefits of voluntary activity.

